# Minimal methylation classifier (MIMIC): A novel method for derivation and rapid diagnostic detection of disease-associated DNA methylation signatures

**DOI:** 10.1038/s41598-017-13644-1

**Published:** 2017-10-18

**Authors:** E. C. Schwalbe, D. Hicks, G. Rafiee, M. Bashton, H. Gohlke, A. Enshaei, S. Potluri, J. Matthiesen, M. Mather, P. Taleongpong, R. Chaston, A. Silmon, A. Curtis, J. C. Lindsey, S. Crosier, A. J. Smith, T. Goschzik, F. Doz, S. Rutkowski, B. Lannering, T. Pietsch, S. Bailey, D. Williamson, S. C. Clifford

**Affiliations:** 1 Wolfson Childhood Cancer Research Centre, Northern Institute for Cancer Research, Newcastle University, Newcastle upon Tyne, UK; 2 Northumbria University, Newcastle upon Tyne, UK; 3 Agena, Hamburg, Germany; 4 NewGene, Newcastle upon Tyne, UK; 5 Department of Neuropathology, University of Bonn Medical Center, Bonn, Germany; 6 Institut Curie and University Paris Descartes, Paris, France; 7 University Medical Center Hamburg-Eppendorf, Hamburg, Germany; 8 Department of Pediatrics, University of Gothenburg and the Queen Silvia Children’s Hospital, Gothenburg, Sweden; 9 Present Address: Queen’s University,, Belfast, BT7 1NN UK

## Abstract

Rapid and reliable detection of disease-associated DNA methylation patterns has major potential to advance molecular diagnostics and underpin research investigations. We describe the development and validation of minimal methylation classifier (MIMIC), combining CpG signature design from genome-wide datasets, multiplex-PCR and detection by single-base extension and MALDI-TOF mass spectrometry, in a novel method to assess multi-locus DNA methylation profiles within routine clinically-applicable assays. We illustrate the application of MIMIC to successfully identify the methylation-dependent diagnostic molecular subgroups of medulloblastoma (the most common malignant childhood brain tumour), using scant/low-quality samples remaining from the most recently completed pan-European medulloblastoma clinical trial, refractory to analysis by conventional genome-wide DNA methylation analysis. Using this approach, we identify critical DNA methylation patterns from previously inaccessible cohorts, and reveal novel survival differences between the medulloblastoma disease subgroups with significant potential for clinical exploitation.

## Introduction

Altered DNA methylation patterns have emerged as a common feature of disease pathogenesis, showing clear potential in diagnostics, sub-classification and prediction of therapeutic response/ disease course^1–7^. In contrast to current high-throughput, genome-wide research methodologies (e.g. whole-genome bisulfite sequencing^8^, DNA methylation arrays^9^), particular challenges exist in the clinical application of disease-associated methylation patterns. These include derivation and validation of representative DNA methylation signatures from genome-scale datasets, and their assessment using platform-independent assays that can be applied rapidly to single samples, including low quality and/or quantity biopsies, in routine diagnostics. To address this, we have developed and validated MIMIC (minimal methylation classifier), a novel polymerase chain reaction (PCR)-based assay for the multiplexed assessment of bisulfite-induced methylation-dependent DNA sequence changes^10^ at multiple signature CpG loci. Sequence-specific single-base variants are exposed by primer extension and, here, coupled to detection by MALDI-TOF mass spectrometry commonly used in clinical DNA diagnostics^11^, enabling the investigation of samples that were not suitable for analysis using existing methods.

We focussed assay development on medulloblastoma, the most common malignant brain tumour of childhood^12^, where DNA methylation signatures have clear potential for use in routine clinical sub-classification^13,14^. Medulloblastoma comprises four primary molecular subgroups – WNT, SHH, Grp3 and Grp4 - defined by distinct methylomic, transcriptomic and genomic features^13–15^. These subgroups display characteristic clinical features, drug targets and outcomes, and have significantly contributed to the 2016 World Health Organisation (WHO) classification of brain tumours^16^. Following design and validation of a MIMIC assay for molecular subgrouping, we assessed its efficacy in limited archival tumour biopsies previously refractory to subgrouping using current research methods, taken from the pan-European HIT-SIOP-PNET4 medulloblastoma clinical trial (2000–2006)^17,18^. This trial enrolled patients negative for all established clinico-molecular risk-factors (termed ‘standard-risk (SR)’ disease^12^), a group for which there is an urgent unmet need to develop biomarker-driven treatment strategies.

## Results

### Derivation of minimal DNA methylation signatures to identify the four medulloblastoma molecular subgroups

**Figure 1:**
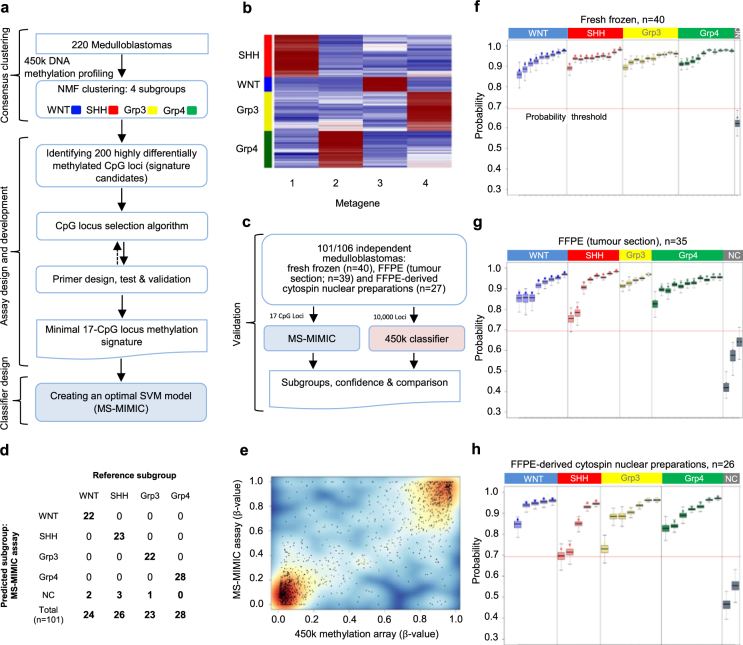
MS-MIMIC, a novel assay to assess minimal DNA methylation signatures, and its application in a proof of concept study for the identification of molecular disease subgroups in medulloblastoma. (**a**) Summary of assay design and development including derivation and validation of minimal DNA methylation signatures. (**b**) Non-negative matrix factorisation (NMF) consensus clustering identified four molecular subgroups (WNT, SHH, Grp3 and Grp4), defined by four metagenes. (**c**) Assessing MS-MIMIC performance against 450k DNA methylation microarray using 101/106 independent medulloblastoma samples. 5/106 samples failed QC (>6/17 CpG locus fails). (**d**) Subgroup classification from MS-MIMIC showed total concordance with the reference subgroup (from 450k and GoldenGate DNA methylation arrays and *CTNNB1* mutation status) after applying a classification confidence probability threshold (NC –non classifiable). (**e**) Estimates of methylation (β-values) using MS-MIMIC assay correlated closely with gold-standard 450k DNA methylation microarray (n = 91). (**f**–**h**) Subgroup assignment and probability estimates (dots) along with 95% confidence intervals (boxplots) for 101 medulloblastoma samples for which DNAs were derived from three types of materials: Fresh frozen biopsies (n = 40), formalin-fixed paraffin-embedded biopsies (FFPE, tumour section; n = 35) and FFPE-derived cytospin nuclear preparations (n = 26). Samples which did not exceed a classification confidence probability threshold of 0.69 (red-line; empirically derived, Supplementary Fig. 5) were deemed nonclassifiable.

**Figure 2:**
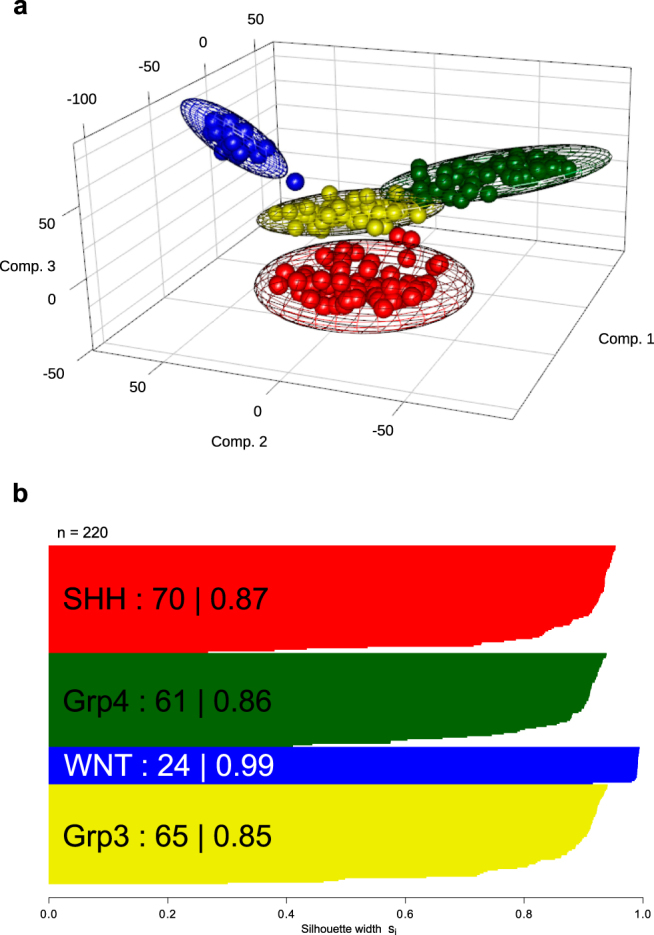
Derivation of the four consensus medulloblastoma molecular subgroups in a training cohort (n = 220) using genome-wide Illumina 450k DNA methylation microarray data. (**a**) Principal Component Analysis (PCA) visualization of groups identified using consensus NMF clustering. Subgroup members are shown in their consensus colours (blue (WNT); red (SHH); yellow (Grp3) and green (Grp4)). Covariance spheroids were plotted at 95% confidence intervals. (**b**) Silhouette plot demonstrates robustness of each group (number and average silhouette width are shown).

**Figure 3:**
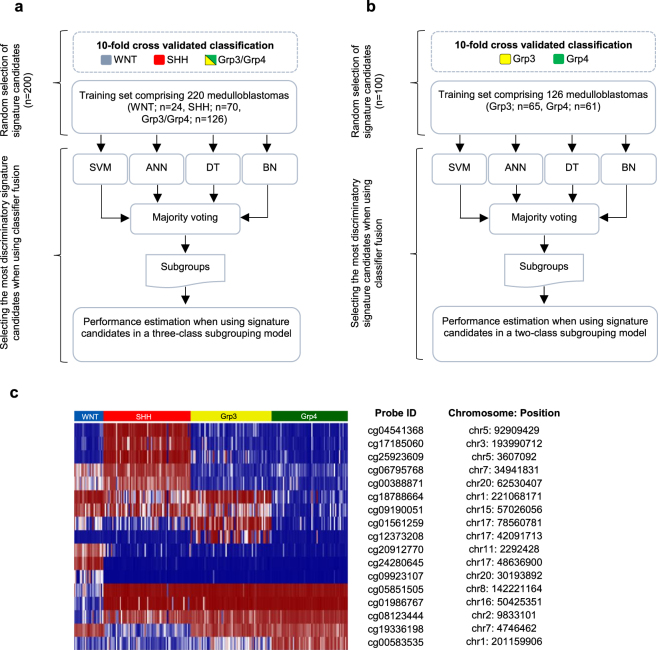
Derivation of a minimal, multiply-redundant, methylation signature for medulloblastoma subgroup identification. Using a three-class (**a**) and two-class (**b**) subgrouping model, a minimal multiply-redundant methylation signature was identified by selecting a subset of signature candidates with the highest ranking in a classifier fusion model (support vector machine (SVM), artificial neural network (ANN), decision tree (DT) and Bayesian network (BN). (**c**) Heatmap of the most discriminatory signature candidates (n = 17) recapitulates the four known medulloblastoma subgroups. Chromosomal locations (hg19 assembly) and identifiers of the 17 CpG loci are reported in the adjacent table.

We first identified a DNA methylation signature of 17 CpG loci, established detection methods and developed a Support Vector Machine (SVM) classification model for distinction of the four medulloblastoma molecular subgroups (WNT, SHH, Grp3 and Grp4; Fig. 1a). Non-negative matrix factorisation (NMF) consensus clustering^13,19,20^ was used to identify subgroup membership of a training cohort comprising genome-scale Illumina 450k DNA methylation microarray data for 220 medulloblastomas (Fig. 1b; Fig. 2). The 50 most discriminatory CpG loci for each subgroup (*i.e*. 200 in total) were considered as signature candidates. These were triaged using (i) a 10-fold cross validated classification fusion algorithm, (ii) a reiterative primer design process where amenability to primer design and multiplex bisulfite PCR was assessed *in silico* (Supplemental experimental methods), and (iii) *in vitro* PCR validation (Fig. 1a; Fig. 3). Candidate signature CpG loci were assayed by the development of a novel application of the Agena iPlex assay^21^, whereby methylation-dependent SNPs representative of CpG methylation status were induced by initial treatment of DNA with sodium bisulfite^10^, followed by multiplexed PCR and single base extension of probe oligonucleotides. The resultant products were quantified by MALDI-TOF MS (Matrix-assisted laser desorption/ionization-time of flight mass spectrometry; Supplementary Fig. 1). The accuracy and precision of methylation estimates from multiplexed extension reactions were tested using incremental proportions of bisulfite-treated methylated:unmethylated DNA (Supplementary Fig. 2). Using these techniques, our optimal, multiply-redundant 17-CpG locus signature was generated. Finally, the training cohort was used to generate an optimised SVM classifier for the signature using 450k DNA methylation array data.

### Development and validation of MS-MIMIC

We next assessed mass spectrometry-minimal methylation classifier (MS-MIMIC) performance against Illumina 450k methylation microarrays in an independent validation cohort of 106 medulloblastoma DNA samples (Fig. 1c) containing all four medulloblastoma subgroups (Supplementary Table 1). DNAs were derived from tissue samples reflecting different clinical fixation methods; fresh-frozen biopsies (n = 40), formalin-fixed paraffin-embedded biopsies (FFPE, tumour section; n = 39), or FFPE-derived cytospin nuclear preparations^18^ (n = 27). QC measures for CpG locus-specific assay failure were established; up to 6 failed CpGs per sample were tolerated within the multiply-redundant signature/classifier without impacting performance and this was the case for 101/106 validation cohort samples (Supplementary Figs 3 and 4). A probability threshold for confident subgroup classification was derived empirically, below which samples were deemed non-classifiable (6/101 samples; 5.7%) (Supplementary Fig. 5). Respective subgroup classifications were compared; MS-MIMIC classifications showed complete concordance with the reference subgroup (as determined by DNA methylation array and/or *CTNNB1* mutation status for WNT tumours^15^; Fig. 1d). Furthermore, CpG-level methylation estimates (β-values) were equivalent between methods (R^2^ = 0.79, p < 0.00001; Fig. 1e). As expected, fresh-frozen derivatives performed best (n = 39/40; 98% successfully subgrouped), with 92% success (n = 56/61) using FFPE-derived DNA (Fig. 1f–h).

### Application of MS-MIMIC to the HIT-SIOP-PNET4 clinical trials cohort

**Figure 4:**
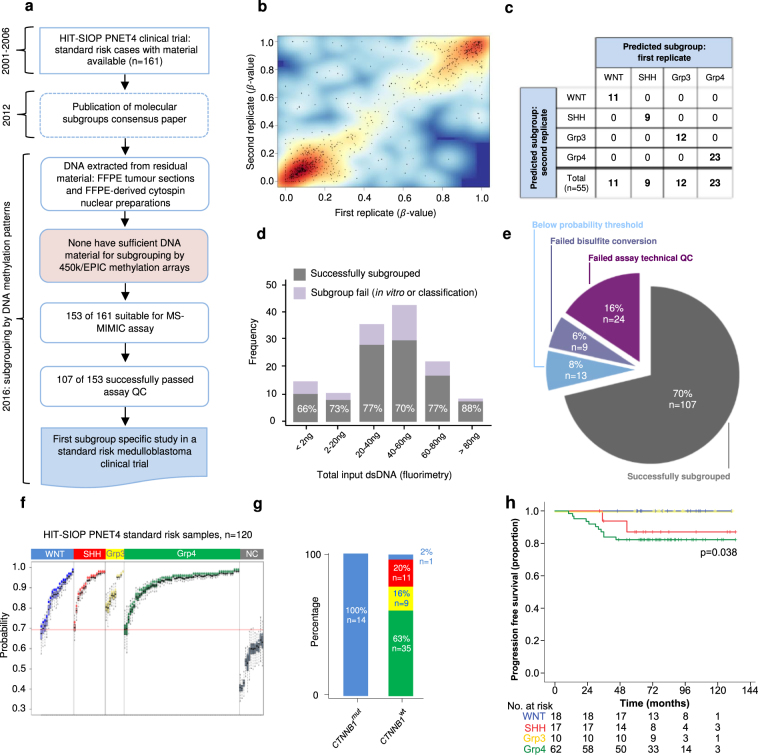
Application of MS-MIMIC to remnant, archival materials from the HIT-SIOP-PNET4 clinical trial cohort enables the first subgroup-specific characterisation of standard-risk medulloblastoma. (**a**) The HIT-SIOP-PNET4 clinical trial ran prior to the discovery of medulloblastoma molecular subgroups (2000–2007) and remnant tumour sample materials available were old, scant and unsuitable for array-based DNA methylomic subgrouping. (**b**,**c**) First and second replicates of individual samples show concordance both at the level of estimate of methylation (β-value; R^2^ = 0.59; n = 55 replicates, 17 CpG loci) and subgroup assigment calls (55/55 replicates; 100%). (**d**) Bar plot of MS-MIMIC assay input dsDNA amounts. The proportions of samples that were successfully subgrouped are shown grey and were consistent across different dsDNA input quantities. Lilac represents samples for which subgrouping was not possible due to QC failure *in vitro* or in classification. (**e**) Proportion of attempted samples (153/161; 95%) successfully subgrouped (107/153; 70%) or failing the assay due to failure to 1) bisulfite convert (9/153; 6%) 2) meet QC citerion for CpG-locus specific failure (>6/17 CpG-locus fails, 24/153 samples; 16%), 3) meet probablility threshold in classification confidence (13/153; 8%). (**f**) Subgroup assignment and probability estimates (dot) along with 95% confidence intervals (boxplots) for 120 standard risk HIT-SIOP-PNET4 tumours after applying the confidence probability threshold (red-line). (**g**) All samples which were *CTNNB1* mutated (*CTNNB1* ^*mut*^; a well-established marker of WNT medulloblastoma) were assigned as WNT by MS-MIMIC. No non-WNT tumours were *CTNNB1* ^*mut*^. (**h**) Progression free survival (PFS) Kaplan-Meier curves for MS-MIMIC derived subgroups reveal that standard-risk Grp4 medulloblastomas show a significantly worse disease outcome compared to other subgroups (p = 0.038, log-rank test). Numbers below x-axis represent patients at risk of event.

Following successful assay development and validation, we next wished to test the application of MS-MIMIC methylation signature detection in limited, poor quality, archival, clinical biopsies. Analysis of remnant material from the HIT-SIOP-PNET4 cohort offers the first opportunity to determine the potential utility of molecular subgroup status to predict disease outcome in a clinical trial of risk-factor negative (SR) medulloblastoma (Fig. 4a, Supplementary Table 2). Only FFPE sections (n = 42/153 available tumour samples) and cytospin nuclear preparations (approximately 30,000 nuclei isolated and centrifuged onto microscope slides^18^; n = 111/153) remained, whose DNA derivatives all fell below quality and quantity thresholds (>200 ng double-stranded DNA (dsDNA)) required for methylation profiling using conventional research methods (Illumina 450k and MethylationEpic arrays^14^). Using MS-MIMIC, 70% (107/153) of samples were successfully subgrouped, and subgroup assignments and β-value estimations were consistent across duplicate determinations (Fig. 4b,c). Assay performance was equivalent across the input DNA range (<2 ng (limit of detection) to 100ng dsDNA, 41.4ng median DNA input, p = 0.852, chi-squared test) (Fig. 4d,e,f). Reasons for assay failure included unsuccessful bisulfite conversion/PCR (6%; 9/153), and inability to classify due to assay QC failure (24%; 37/153) (Fig. 4e). These findings from HIT-SIOP-PNET4 reveal important subgroup-dependent molecular pathology in SR medulloblastoma. Grp4 was most common (n = 62; 58%), with approximately equivalent numbers of WNT (18/170; 16%), SHH (17/107; 16%) and Grp3 (10/107; 9%) tumours observed. As expected, all tumours with *CTNNB1* mutations (n = 14) were classified as WNT (Fig. 4g). The majority of events (defined as disease recurrence or progression following treatment) observed (11/13) affected Grp4 patients (82% 5-year progression-free survival (PFS)), with > 95% PFS in non-Grp4 patients (p = 0.038, log-rank test; Fig. 4h). Subgroup assignment will thus be essential to inform future clinical and research studies in SR medulloblastoma.

## Discussion

We have provided a blueprint for defining minimal, multiply-redundant disease-associated DNA methylation signatures from genome-wide datasets, and have developed MS-MIMIC as a validated assay for their assessment, including open-source classification tools for data interpretation (http://medulloblastomadiagnostics.ncl.ac.uk; Supplementary Fig. 6). Unlike research methodologies (e.g. Illumina 450k and MethylationEPIC arrays) which require batched assessments (≥8 samples per run), MS-MIMIC exploits detection technologies in common clinical use (MALDI-TOF) to enable rapid (<3 days from DNA extraction to result), low-cost (<$200 per sample in 2017), routine assessment in single or multiple samples. The assay format allows a flexible, modular approach, in which multiplex PCRs can be straightforwardly added or removed, offering the ability to adapt or extend panels to evolving clinical needs. Moreover, its low DNA input requirements and applicability to archival sample collections has the potential to unlock previously inaccessible molecular information from informative cohorts, as demonstrated for HIT-SIOP-PNET4. This assessment of DNA methylation signatures in the clinical setting holds rich promise for molecular sub-classification and prognostication across diverse diseases.

## Methods

### Cohorts and sample collection

Three cohorts (training; n = 220, validation; n = 106 and test; n = 153) were used in this study and are described in Supplementary Table 1. The training and validation cohorts comprised archival non-trial medulloblastoma DNA samples and included tumour samples provided by the UK Children’s Cancer and Leukaemia Group (CCLG) biobank as part of CCLG-approved biological study BS-2007–04. The validation cohort consisted of samples with varying DNA quality, to assess assay performance. The test cohort included samples from the HIT-SIOP-PNET4 clinical trial (2001–2006)^17^. All tumours assayed had a confirmed histopathological diagnosis of medulloblastoma, with a high tumour cell content. Informed consent was obtained from all participants and/or their legal guardians. All experiments were performed in accordance with relevant guidelines and regulations.

### Identification of a minimal methylation signature for discrimination of medulloblastoma molecular subgroups

Non-negative matrix factorisation (NMF) consensus clustering^19^ was used to identify the four recognised medulloblastoma consensus molecular subgroups^13^ using 220 training cohort samples run on the Illumina 450k methylation microarray platform (Fig. 2). The 50 most differentially-methylated CpG loci for each subgroup were selected as potential signature candidates using limma^22^. An iterative CpG locus selection algorithm was used to select signature gene candidates. To optimise signature loci redundancy in each level, up to 6 loci were repeatedly removed at random and classification performance evaluated. The 17 signature loci with the highest ranking in classification were identified (Fig. 3).

### Assay methodology

The MS-MIMIC assay is based on an adaptation of Agena Biosciences’ iPLEX assay and the MassARRAY platform^21^. In order to determine methylation status at each signature CpG locus, bisulfite treatment of DNA was used to induce methylation-dependent SNPs. These regions were amplified by multiplex PCR, followed by single base extension using mass-modified dideoxynucleotide terminators. MALDI-TOF mass spectrometry then identifies the proportions of the induced-SNP alleles, from which methylation status can be inferred.

### Primer design and validation

PCR and extension primers were designed for multiplex assessment of methylation in 17 signature loci (Supplemental experimental methods) across three multiplexes. Plex 1 contained an additional bisulfite conversion control, targeting an invariably unmethylated locus which undergoes complete conversion to uracil. The multiplexes were validated *in vitro* using a triplicate mixture series of control DNAs ranging from 0–100% methylation (Supplementary Fig. 2). The correlation between the input and estimated DNA methylation was calculated, and amplicons with a poor correlation were discarded and replaced with a new CpG locus as part of the iterative redesign process (Figs 1a and 3). All signature loci had good linear correlation (average correlation coefficient R^2^ = 0.86.)

### Assay implementation

Where possible, 100ng of DNA was bisulfite converted and purified using the Qiagen EpiTect Bisulfite kit, according to manufacturer’s protocol. To ensure that template was not too fragmented for analysis, a test bisulfite PCR, targeting a 200 bp amplicon, was performed (Supplementary Table 3). Reaction mixtures and thermal cycling parameters used to amplify the 17 signature loci in multiplex are shown in the Supplementary Table 4. Successful amplification was confirmed by gel electrophoresis. The multiplex primer iPLEX extension assay was performed as previously described^23^. Primer sequences for multiplex signature loci PCR and iPLEX extension PCR are shown in the Supplementary Table 5 and 6. Mass spectra for the multiplexes were acquired on a MALDI-TOF mass spectrophotometer (Voyager DE; PerSeptive Biosystems).

### Classifier design and validation

Two support vector machine (SVM) classifier models were created to assign subgroup and corresponding probability^24^, one trained on 450k array data using the 10,000 most variably methylated CpG loci, the second with the 17-CpG locus signature from the training cohort (Fig. 1c). Subsequently, 101/106 validation cohort samples were used to assess MS-MIMIC concordance with 450k-derived data, at the level of molecular subgroup call (Fig. 1d) and estimates of methylation β-value (Fig. 1e). It was anticipated that when applying MS-MIMIC to poor quality samples, certain loci would not be assessable. Using bootstrapped datasets (n = 10,000), a threshold of 6 was established for a maximum acceptable number of missing loci. Missing loci were imputed using expectation maximisation. Subgroup assignments using MS-MIMIC classifier were compared against corresponding subgroup calls from the 450k classifier. A threshold for probability of assignment by the MS-MIMIC classifier was empirically set to 0.69, below which samples were non-classifiable (Supplementary Fig. 5).

### Application to HIT-SIOP-PNET4 clinical trial cohort

Following successful assay development and validation, we applied MS-MIMIC to remnant, poor quality, archival, biopsies from the HIT-SIOP-PNET4 clinical trial of risk-factor negative medulloblastoma (Fig. 4a, Supplementary Table 2). Only FFPE sections (n = 42/153 available tumour samples) and cytospin nuclear preparations (approximately 30,000 nuclei isolated and centrifuged onto microscope slides^18^; n = 111/153) remained, whose DNA derivatives all fell below quality and quantity thresholds (>200 ng double-stranded DNA (dsDNA)) required for methylation profiling using conventional research methods (Illumina 450k and MethylationEpic arrays^14^). We assessed differential survival of the MS-MIMIC subgroup assignments using log-rank tests.

Further technical details are provided in the Supplemental experimental methods.

### Data availability

The datasets generated during and/or analysed during the current study are available from the corresponding author on reasonable request.

## Electronic supplementary material

Supplementary information
