# Supplementary material for: Minimal methylation classifier (MIMIC): A novel method for derivation and rapid diagnostic detection of disease-associated DNA methylation signatures
Source: Sci Rep. 2017 Oct 18;7:13421. doi: 10.1038/s41598-017-13644-1 (PMC5647382; doi:10.1038/s41598-017-13644-1)
Supplement: Supplementary file 1 — Supplementary information [file 41598_2017_13644_MOESM1_ESM.doc]

**Minimal methylation classifier (MIMIC): A novel method for derivation and rapid diagnostic detection of disease-associated DNA methylation signatures**

Schwalbe, E.C.^1,2†^, Hicks, D.^1†^ , Rafiee, G.1^†‡^, Bashton, M.^1^, Gohlke, H.^3^, Enshaei, A.^1^, Potluri, S.^1^, Matthiesen, J.^1^, Mather, M.^1^, Taleongpong, P.^1^, Chaston, R.^4^, Silmon, A.^4^, Curtis, A.^4^, Lindsey, J.C.^1^, Crosier, S.^1^, Smith, A.J.^1^, Goschzik, T.^5^, Doz, F.^6^, Rutkowski, S.^7^, Lannering, B.^8^, Pietsch, T.^5^, Bailey, S.^1^, Williamson, D.^1^, Clifford, S.C*.^1^

^1^Wolfson Childhood Cancer Research Centre, Northern Institute for Cancer Research, Newcastle University, Newcastle upon Tyne, U.K.

^2^Northumbria University, Newcastle upon Tyne, U.K.

^3^Agena, Hamburg, Germany

^4^NewGene, Newcastle upon Tyne, U.K.

^5^Department of Neuropathology, University of Bonn Medical Center, Bonn, Germany

^6^Institut Curie and University Paris Descartes, Paris, France

^7^University Medical Center Hamburg-Eppendorf, Hamburg, Germany

^8^Department of Pediatrics, University of Gothenburg and the Queen Silvia Children's Hospital, Gothenburg, Sweden

†These authors contributed equally to this work. * Corresponding author.

‡Present address; Queen’s University Belfast, U.K.


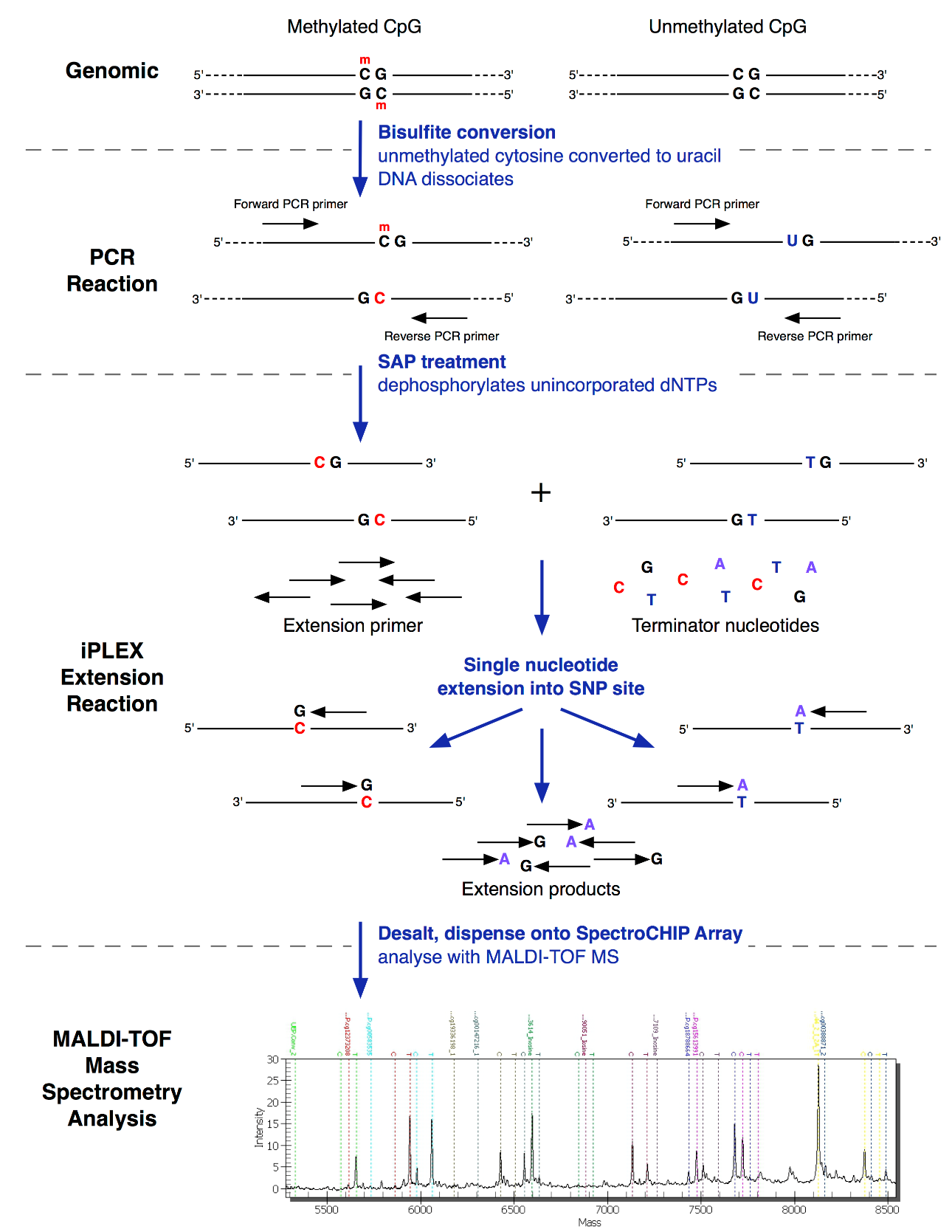


**Supplementary Figure 1. MS-MIMIC assay enables identification of methylation status at multiple loci.** The signature loci constitute 17 variably methylated CpG dinucleotides; the methylation status of which can be used to assign molecular subgroup. The Agena iPLEX assay was adapted to incorporate a bisulfite conversion step, inducing methylation-dependent SNPs that are subsequently detected using mass spectrometry based SNP-typing. Figure adapted from iPLEX application note (Agena Bioscience, San Diego, CA).


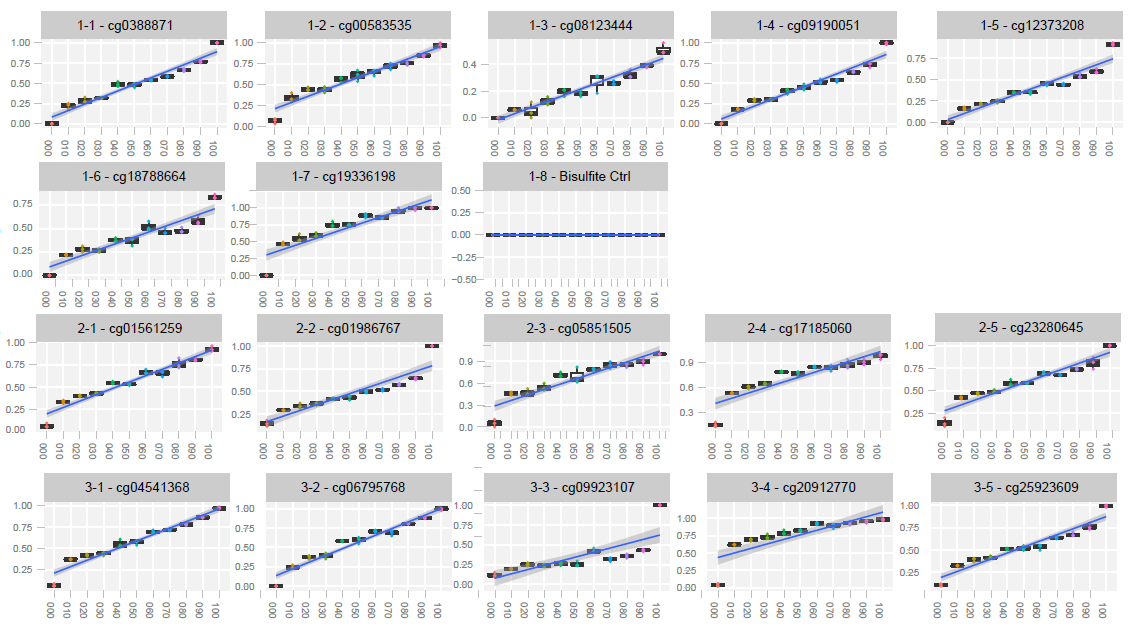


Plex1

Plex2

Plex3

Fraction methylated DNA

Estimated methylation; MS-MIMIC

**Supplementary Figure 2. *In vitro* validation demonstrates strong correlation between true methylation and estimates of methylation made using MS-MIMIC.** Graphs show comparison between incremental ratios of methylation from 0 to 1 (in 0.1 intervals, x axis) and MS-MIMIC estimates (β-value, y axis; each increment was run in triplicate). The methylation status of the 17 signature loci was assayed in multiplex; Plex 1-3 as indicated. Plex 1 contains an additional bisulfite conversion control (Plex1-8 – bisulfite ctrl), which is invariably unmethylated and undergoes complete conversion to uracil.

Fresh frozen

FFPE (tumour section)

FFPE-derived cytospin nuclear preparations

Frequency

60

40

20

0


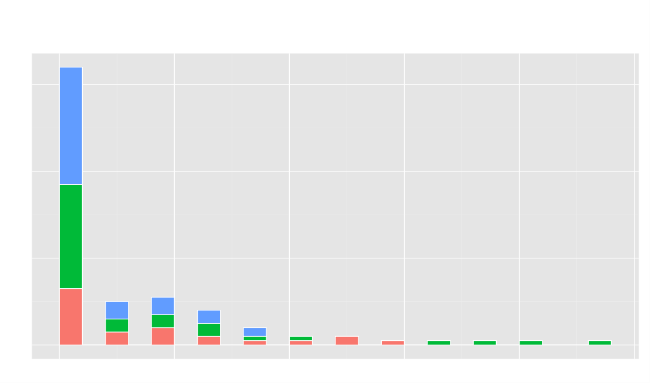


0 1 2 3 4 5 6 7 8 9 1011 12

Number of missing β-values

**Supplementary Figure 3. The majority of validation cohort samples return complete sets of 𝛃-values, even for FFPE tumour material.** Bar plot shows the number of missing β-values per sample in the validation cohort (n=106) after MS-MIMIC assay. 63/106 (59%) samples reported complete sets of β-values whereas 5/106 (5%) samples had more than 7 missing β-values (QC measure for CpG locus-specific threshold; black line). Bars are stacked according to method of tissue fixation (fresh frozen, blue; FFPE (tumour section), green; FFPE-derived cytospin nuclear preparations, pink).

No

Yes

Random missing pattern generator:

generating 1000 bootstrap samples (without replacement) with 1,…,n missing positions on 450k DNA array data

β-values of missing positions are replaced by *g*(β)

Subgrouping 1000 bootstrap samples

n=n+1

Assessment of confusion matrix, calculation of accuracy and confidence interval

n=1

n≤10

Average of AUC (%)

**a**

**b**

Input

(β-value)Output

(*g*(β))β < 0.25

or β > 0.751-β 0.25 <β < 0.510.5 ≤β ≤ 0.750

Average of AUC (%)

80

90

100

70

60

50


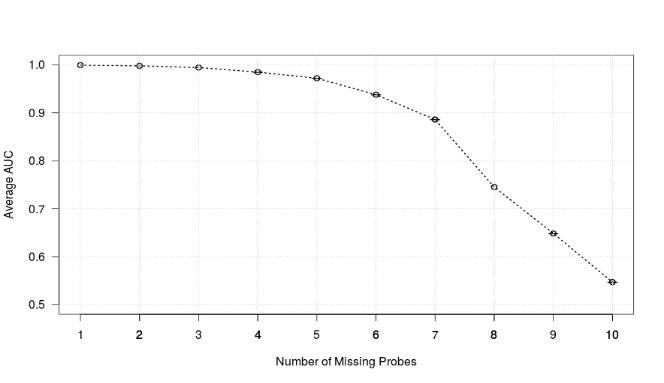


6

1

2

3

4

5

7

8

9

10

**c**

Number of replaced β-values

**Supplementary Figure 4. Empirical determination of the maximal number of permissible missing**
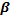
**-values. a)** The prediction accuracy of the SVM classifier model was evaluated *in silico* by replacing missing data with confounding methylation values, using the transformation shown in **(b)**. **(c).** Using the 17-locus signature from 450k DNA methylation array data, random combinations of 1 to 10 β-values were replaced with confounding data and the performance of the classifier assessed. The average area under curve (AUC) from 1000 bootstraps was plotted. An average AUC of > 94% is achieved up to 6 missing β-value data points. Assay performance declines with more than 6 missing β-value data points (QC threshold; blue dotted line).

Probability Density Function (PDF)

Final probability estimates

**Supplementary Figure 5. Derivation of a QC threshold for confident subgroup classification.** Histogram of the final probability estimates of subgroup assignment in the validation cohort and the fitted beta distribution (PDF; red line), using the moment matching estimation technique (MME). The blue dotted line indicates the 5^th^ percentile of the beta distribution. When the probability estimate falls below 0.69, the sample is considered non-classifiable (NC).

**a**


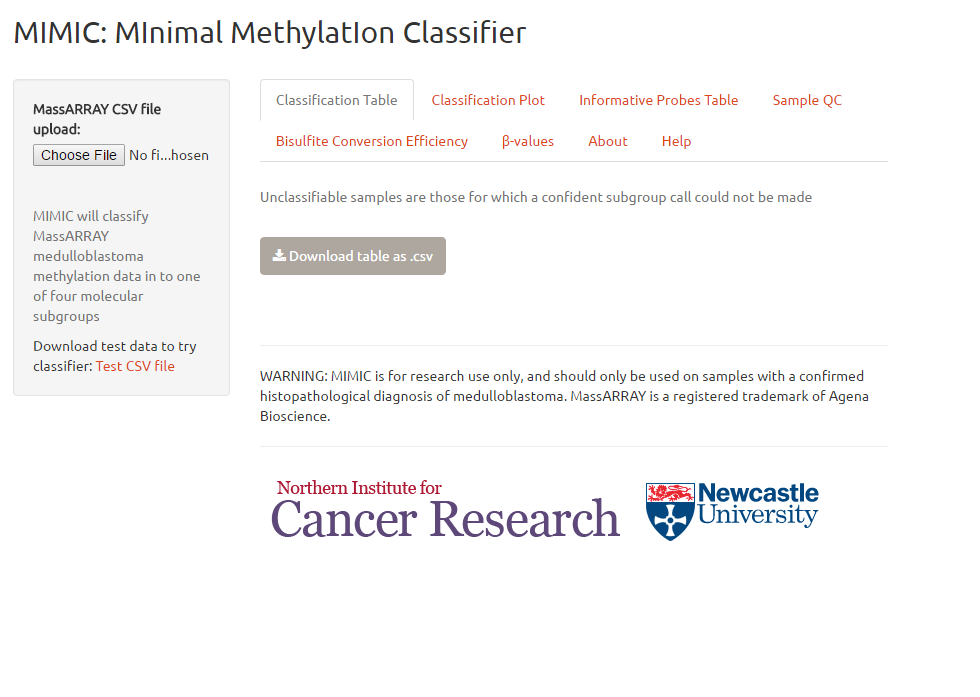


**b**


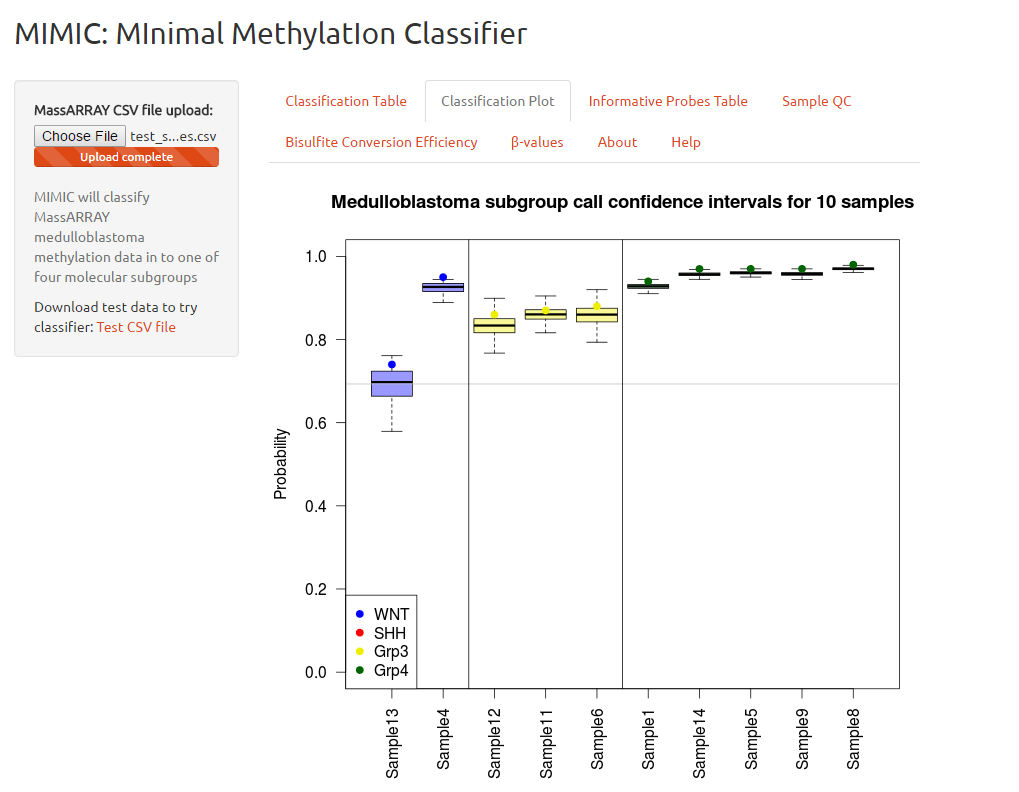


**c**


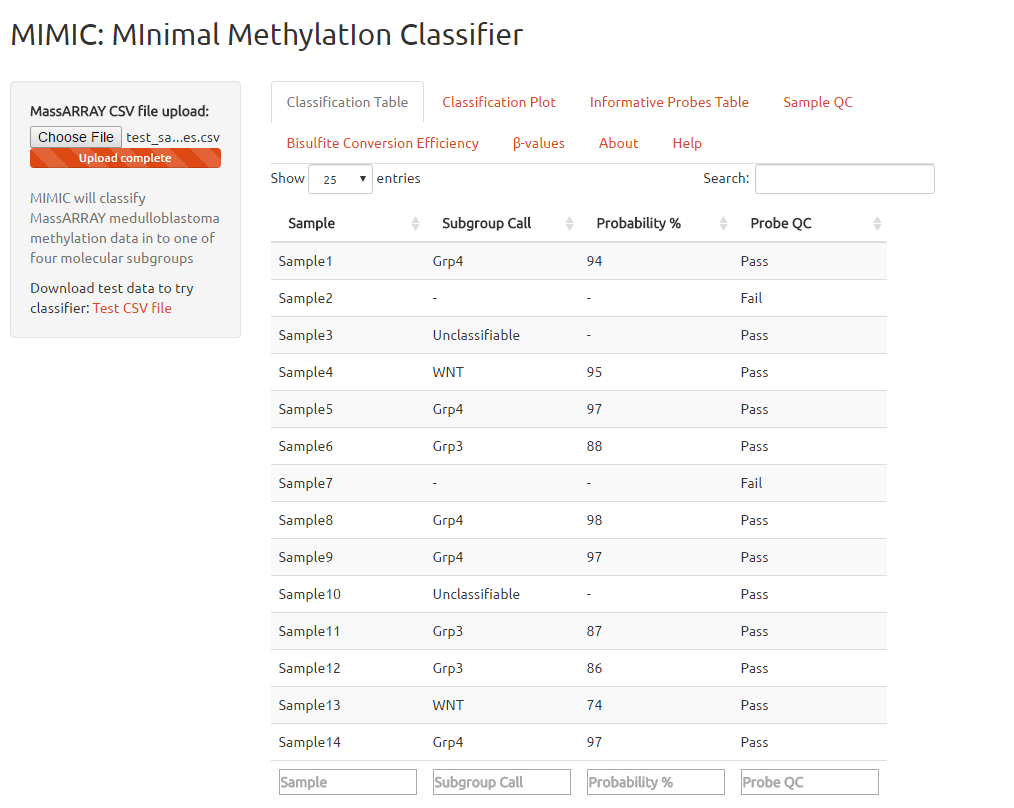


**d**


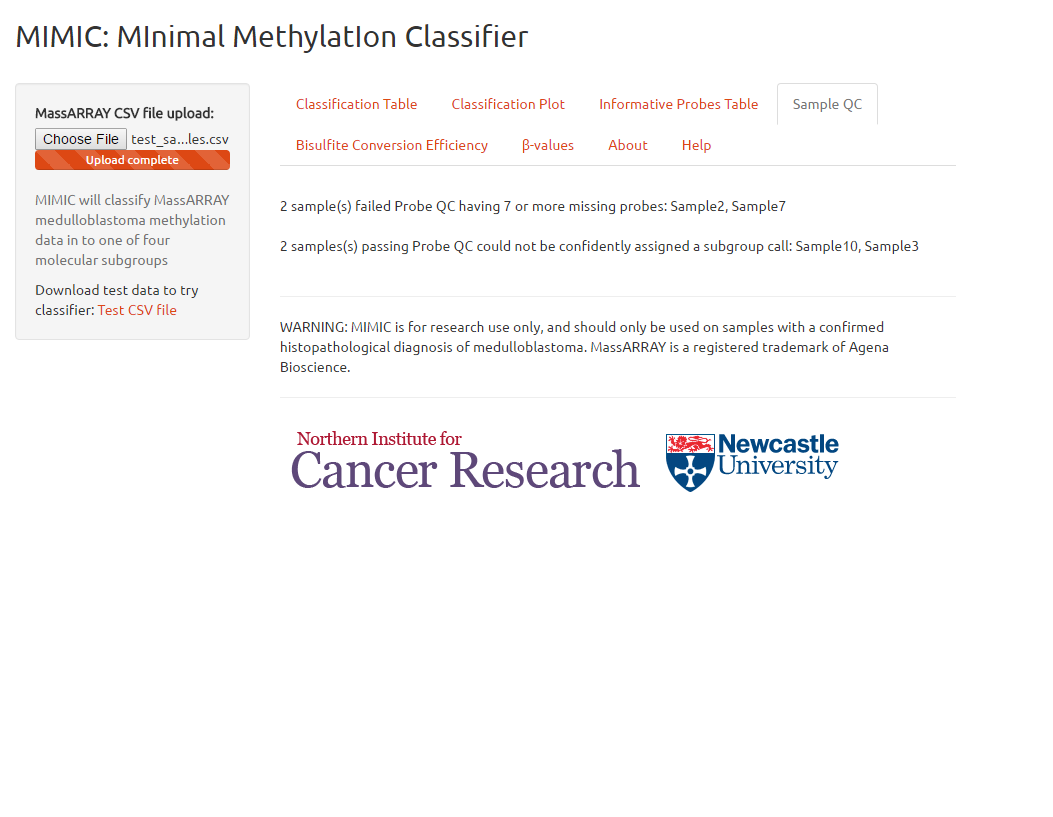


**Supplementary Figure 6. Medulloblastoma subgroup classification web app pages (**[**http://medulloblastomadiagnostics.ncl.ac.uk**](http://medulloblastomadiagnostics.ncl.ac.uk/)**). (a)** MIMIC main page which provides a test input file. **(b)** Classification plot demonstrating subgroups and confidence intervals. **(c)** Classification table showing subgroup call, assigned probabilities and QC results for the test input file. **(d)** Sample QC details.


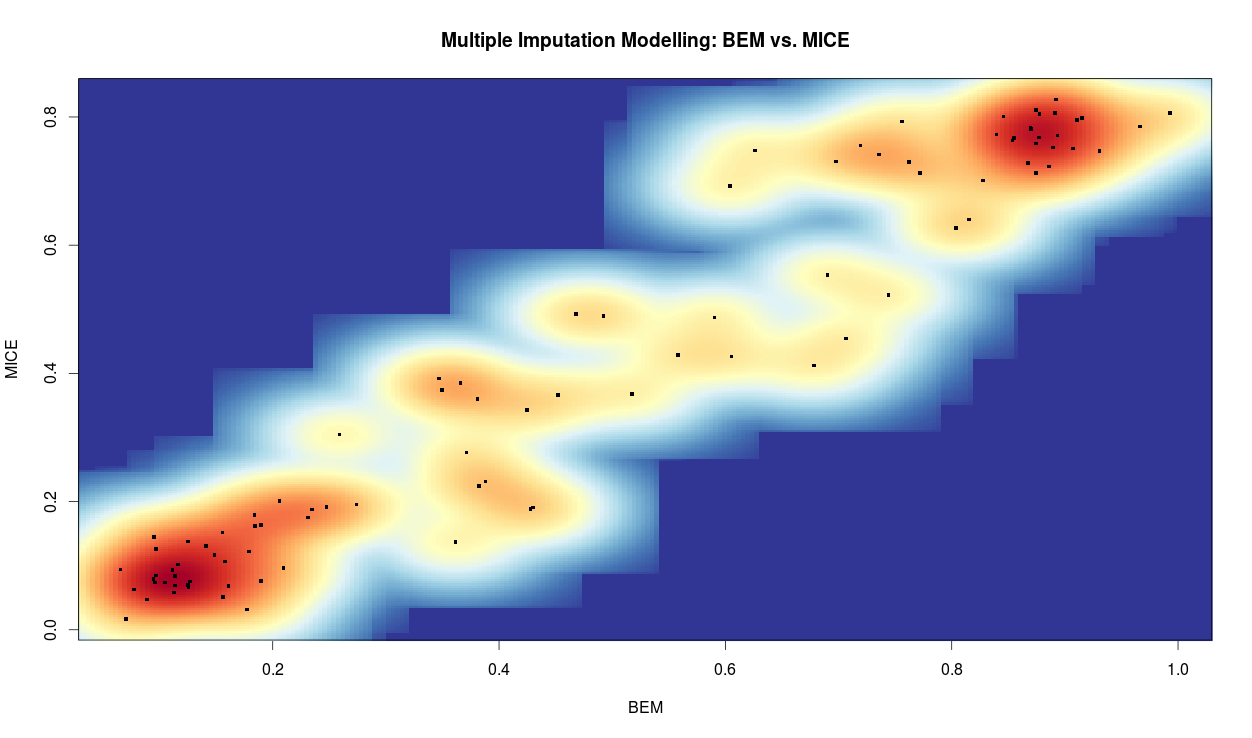


Bootstrapped-based expectation maximization (BEM)

Multivariate imputation by chained equations (MICE)

**Supplementary Figure 7**. **Predicted subgroup is insensitive to multiple imputation modelling technique.** Scatterplot of β-values generated by the bootstrapped-based expectation maximization (BEM) (x axis) and multivariate imputation by chained equations (MICE) (y axis) showing a strong correlation between the two methods (R^2^=0.77).

| **Cohort** | **Purpose** | **Available tumour material** | **Molecular subgroup** | | | | |
| --- | --- | --- | --- | --- | --- | --- | --- |
|  |  |  | **WNT** | **SHH** | **Grp3** | **Grp4** | **Total** |
| 450k DNA methylation array cohort^1^ | Training | Fresh Frozen or FFPE block | 24 | 70 | 65 | 61 | 220 |
| MS-MIMIC cohort (n=106)^2^ | Validation | Fresh Frozen | 9 | 11 | 10 | 10 | 40 |
|  |  | FFPE (tumour section) | 10 | 12 | 7 | 11 | 39 |
|  |  | FFPE-derived cytospin nuclear preparation | 6 | 6 | 8 | 7 | 27 |
| HIT-SIOP-PNET4 SR cohort (n=161)^3^ | Test | FFPE (tumour section) | NA | NA | NA | NA | 55 |
|  |  | FFPE-derived cytospin nuclear preparation | NA | NA | NA | NA | 106 |

**Supplementary Table 1. Training, validation and test cohorts.** 1) Illumina 450k DNA methylation microarray data for 220 medulloblastomas was used to identify a DNA methylation signature and develop a support vector machine (SVM) model for distinction of the four medulloblastoma molecular subgroups. 2) An independent cohort of medulloblastoma samples containing all four medulloblastoma subgroups and from samples reflecting different clinical fixation methods (fresh-frozen biopsies (n=40), formalin-fixed paraffin-embedded biopsies (FFPE, tumour section; n=39), or FFPE-derived cytospin nuclear preparations (n=27)) was used to assess MS-MIMIC performance against 450k methylation microarrays and to empirically derive assay QC thresholds. For this cohort, molecular subgroup was assigned by either 1) 450k DNA methylation microarray (90%, 95/106), 2) Goldengate DNA methylation microarray (7%, 8/106) or for WNT subgroup only, confirmed *CTNNB1* mutation (3%, 3/106). 3) The HIT-SIOP-PNET4 standard-risk (SR) cohort was used to ascertain the applicability of MS-MIMIC to poor quality, archival DNA, and assess the significance of molecular subgroups in SR medulloblastoma. During sample processing for reference pathology, excess tumour material was collected for biological studies. Remnant archival FFPE tumour sections and FFPE-derived cytospin nuclear preparations (glass slides originally created for interphase fluorescence *in situ* hybridization (iFISH) analysis) were collected where available (n=161) and subjected to MS-MIMIC classification of molecular subgroup.

| **Variable** | **Categories** | **Standard Risk (SR) HIT-SIOP-PNET4 cohort** | | **p-value** |
| --- | --- | --- | --- | --- |
|  |  | **All SR cases**  **n=161** | **SR cases successfully sub-grouped (MS-MIMIC) n=107** |  |
| Gender | Male | 106 (66%) | 63 (59%) | 0.248 |
|  | Female | 55 (34%) | 44 (41%) |  |
| Age at diagnosis  (years) | ≤ 5 | 24 (15%) | 16 (15%) | 0.717 |
|  | 6 to ≤ 10 | 78 (48%) | 56 (52%) |  |
|  | 11 to ≤ 15 | 42 (26%) | 28 (26%) |  |
|  | ≥ 16 | 17 (11%) | 7 (7%) |  |
|  | Mean/Median | 9.6/9.0 | 9.4/8.0 |  |
|  | Min-Max | 4-20 | 4-20 |  |
| Pathological variant | Classic | 131 (81%) | 85 (79%) | 0.696 |
|  | DMB | 30 (19%) | 22 (21%) |  |
| Survival | 5yr EFS | 83±3% | 87±3% | 0.281 |

**Supplementary Table 2. Demographics of the cohort of standard risk (‘SR’) medulloblastoma patients from the HIP-SIOP-PNET4 clinical trial.** The cohort represents all patients with available samples, and a ‘standard-risk’ classification, defined as assessable and negative for all established risk-factors of poor prognosis (metastatic disease, incomplete surgical resection, LCA pathology, *MYC* or *MYCN* amplification). Comparison of the available and subgrouped cohorts did not show significant differences. DMB – desmoplastic medulloblastoma. P values from Fisher’s exact tests of association and log rank (Mantel-Cox) are shown.

| **Reaction components** | |  | **Cycling parameters** | | |
| --- | --- | --- | --- | --- | --- |
| **Reagent** | **Volume (μl)** |  | **Temp**  **(^ο^C)** | **Time** | **# of cycles** |
| Forward primer (10μM)  5’-GGG GTG TTA GGA AGT TTT TGA-3’ | 2 |  | 95 | 10m | 1 |
| Reverse primer (10μM)  5’-CAA CCC CAA ACC AAA CTA TC-3’ | 2 |  | 94 | 50s | 5 |
| 10x AmpliTaq Gold buffer II | 2 |  | 60 | 50s |  |
| 25mM MgCl2 | 1.2 |  | 72 | 50s |  |
| dNTP mix (5mM) | 0.8 |  | 94 | 50s | 40 |
| RNase-free dH_2_0 | 9.8 |  | 55 | 50s |  |
| bisulfite converted DNA | 2 |  | 72 | 50s |  |
| Amplitaq gold DNA polymerase | 0.2 |  | 75 | 5m | 1 |
| Total | 20 |  | 4 | ∞ | 1 |

**Supplementary Table 3. Bisulfite conversion test PCR reaction mixtures and thermal cycling parameters**.

| **Reaction components** | |  | **Cycling parameters** | | |
| --- | --- | --- | --- | --- | --- |
| **Reagent** | **Volume (μl)** |  | **Temp (^ο^C)** | **Time** | **# of cycles** |
| Forward primer mix (10μM) | 1 |  | 95 | 2m | 1 |
| Reverse primer mix(10μM) | 1 |  | 94 | 30s | 45 |
| 10x AmpliTaq Gold buffer II | 2 |  | 56 | 30s |  |
| 25mM MgCl2 | 1.6 |  | 72 | 60s |  |
| dNTP mix (5mM) | 0.2 |  | 72 | 1m | 1 |
| RNase-free dH_2_0 | 2.8 |  | 4 | ∞ | 1 |
| bisulfite converted DNA | 2.0 |  |  | | |
| Amplitaq gold DNA polymerase | 0.4 |  |  |  |  |
| Total | 10 |  |  |  |  |

**Supplementary Table 4. Reaction mixtures thermal cycling parameters for multiplex signature loci PCR.**

| **Reaction components** | |  | **Cycling parameters** | | |
| --- | --- | --- | --- | --- | --- |
| **Reagent** | **Volume (μl)** |  | **Temp (^ο^C)** | **Time** | **# of cycles** |
| H_2_O | 0.74 |  | 94 | 30s | 1 |
| iPLEX Buffer (10x) | 0.2 |  | 94 | 5s | 1 |
| iPLEX termination mix | 0.1 |  | 52 | 5s | 39 |
| EXT Primer mix | 0.94 |  | 80 | 5s |  |
| iPLEX enzyme | 0.02 |  | 72 | 3m | 1 |
| signature loci PCR product | 5 |  |  | | |
| Total | 10 |  |  |  |  |

**Supplementary Table 5. Reaction mixtures thermal cycling parameters for iPLEX extension PCR.**

|  | | **Multiplex signature loci PCR** | | **iPLEX extension PCR** |
| --- | --- | --- | --- | --- |
| **Plex** | **SNP_ID** | **Forward primer sequence** | **Reverse primer sequence** | **Primer sequence** |
|  | Control | ACGTTGGATGGAAGTTGTTTTTGAGTAGGG | ACGTTGGATGACAAAAACAATACTACCCCC | TTT GAG TAG GGT GGT TG |
| 1 | cg12373208 | ACGTTGGATGGTTTTGGGTATTGGTTTTTG | ACGTTGGATGCTCCAATTAACCATTAACACC | GGA AGA TTT TTT TGG GGT |
|  | cg00583535 | ACGTTGGATGTTGGATTGGAGGAGAAGGAG | ACGTTGGATGCCAACTACTACTTACTAAAAC | GGG TAG AGG GAG TAG AGG |
|  | cg19336198 | ACGTTGGATGGGAGTTGGGTTGATTTTT | ACGTTGGATGACCCAACAATTCCAAACTAC | TTG GGT TGA TTT TTT TTG GT |
|  | cg09190051 | ACGTTGGATGGAGTTGTTGGTTGGTGTGG | ACGTTGGATGACCTTTTCACTTTCCAAACC | IGG ATT GTT IGG AAT TTG TAG G |
|  | cg18788664 | ACGTTGGATGGGGAGTGTAGATAGGTTAT | ACGTTGGATGCCCTTTATCTACTACAAACC | TTT GTA AGG GTT TAG ATT TGA TTT |
|  | cg08123444 | ACGTTGGATGGGTAGTTGGAAGTTGTTAAG | ACGTTGGATGCACCCTATCTCTACACCATC | TTT AGG GTT TTT TAA IGG IGG GGT TA |
|  | cg00388871 | ACGTTGGATGGTGAAAGAGGATAAGATTAG | ACGTTGGATGAAACACCACTACCCTCATC | GTG AAA GAG GAT AAG ATT AGG GTT TA |
| 2 | cg05851505 | ACGTTGGATGGGGTTTTAGTTGTTTTAGA | ACGTTGGATGAACCCTACCTCCCAAATCCC | GAG GAA GGT ATG GAG TTT |
|  | cg01561259 | ACGTTGGATGTCCTTCTATACACACAACAC | ACGTTGGATGGGTAAGTAGGTTTTGTATAG | TTA CTA ACI TAA CCC ATA ACC |
|  | cg24280645 | ACGTTGGATGTCACTCACCTTAACCCTACC | ACGTTGGATGGGTAGGAGAGAGTTAGAGT | CCT CIT AAA TTT ATA CTT TCC AC |
|  | cg01986767 | ACGTTGGATGAACCAAAAACACCCCTTCTA | ACGTTGGATGGAGGAGATTGAAAAAAGGTTG | AAC CTC ATC ATT AAT AAC ICT AAC |
|  | cg17185060 | ACGTTGGATGCCCCACAACCATATATATCC | ACGTTGGATGTGGAGGGTAGATTTTGATTG | ACA ACC ATA TAT ATC CAA TCT AAC AC |
| 3 | cg20912770 | ACGTTGGATGAAACCCAACAAAAACCCAAC | ACGTTGGATGGGGGTTAGGGTTTTAGTTAA | TAC TCC CCI TAA ACC AC |
|  | cg25923609 | ACGTTGGATGATCCTACAAACTATCACCCC | ACGTTGGATGGATGAGGGAAGAAGTTAGTT | AAT AAC CTT CCA AAA CIC C |
|  | cg04541368 | ACGTTGGATGTTACACATTCTCTCCTACCC | ACGTTGGATGGGGATTGGTTTTGATGATAG | CIC TTA AAA AAA CAA CIT CCC |
|  | cg09923107 | ACGTTGGATGCCTCTTACCCCCTAAATAAC | ACGTTGGATGGTGTTTTTTGGGGAAGGG | ATC CCT AAA AAA AAC ICT TCA AC |
|  | cg06795768 | ACGTTGGATGGGGTAGATTTAGGTTGGTG | ACGTTGGATGCCTACCTCTCACTATAATTAC | TGT TTT AAA ATT TTA GAG GAG AG |

**Supplementary Table 6. Primer sequences for multiplex signature loci and iPLEX extension PCR**

|  | | **Predicted subgroup:**  **BEM imputation modelling** | | | | |
| --- | --- | --- | --- | --- | --- | --- |
|  |  | **WNT** | **SHH** | **Grp3** | **Grp4** | **NC** |
| **Predicted subgroup:**  **MICE imputation modelling** | **WNT** | **22** | 0 | 0 | 0 | 0 |
|  | **SHH** | 0 | **23** | 0 | 0 | 0 |
|  | **Grp3** | 0 | 0 | **22** | 0 | 0 |
|  | **Grp4** | 0 | 0 | 0 | **28** | 0 |
|  | **NC** | 0 | 0 | 0 | 0 | **6** |
|  | **Total**  **(n=101)** | **22** | **23** | **22** | **28** | **6** |

**Supplementary Table 7. Complete concordance in validation cohort subgroup classification after applying MICE, an alternative imputation modelling technique.**

**SUPPLEMENTAL EXPERIMENTAL METHODS**

**Cohorts and sample collection.**

Three cohorts (training, validation and test) were used in this study and are described in **Supplementary Table 1**. The training and validation cohorts comprised archival non-trial medulloblastoma DNA samples, whereas the test cohort encompassed samples from the HIT-SIOP-PNET4 clinical trial (2001-2006)^17^ for which subgrouping by conventional means was not possible due to limited remaining material. The cohort represents all patients from the trial with available samples and a ‘standard-risk’ (SR) classification, defined as assessable and negative for all established risk-factors of poor prognosis (metastatic disease (M+), incomplete surgical resection (R+), large-cell/anaplastic (LCA) pathology, *MYC* or *MYCN* amplification; **Supplementary Table 2**)^12^ and allowed, for the first time, the assessment of molecular subgroup in a SR clinical trial setting.

**Development of a 17-locus minimal methylation signature for discrimination of medulloblastoma molecular subgroups.**

All the analyses in this section were performed in the R statistical environment^25^ (v3.3.1) and using Weka software^26^ (v3.6.13). Non-negative matrix factorisation (NMF) consensus clustering^19^ was used to identify the four recognised medulloblastoma consensus molecular subgroups^13, 20^ using 220 samples run on the Illumina 450k methylation microarray platform (**Fig. 2).** The 200 most differentially methylated CpG loci were selected using limma^22^ (linear models for microarray data). A two-level (three-class and two-class), iterative CpG locus selection algorithm based on a 10-fold cross validated classification was used. In each level of subgrouping, a classifier fusion model with a majority vote combiner^27^ including SVM (Support Vector Machine), ANN (Artificial Neural Network), DT (Decision Tree) and BN (Bayesian Network) was used to minimise the misclassification for each subset of locus combinations. To optimise signature loci redundancy in each level, up to 6 loci were repeatedly removed at random and classification performance evaluated. The 17 signature loci with the highest ranking in classification were identified (**Fig. 3**).

**Assay methodology**

The MS-MIMIC assay is based on an adaptation of Agena Biosciences’ (formerly Sequenom) iPLEX assay and the MassARRAY platform^21^. In order to determine methylation status at each of the 17 signature CpG loci, bisulfite treatment of DNA was used to induce methylation-dependent single nucleotide polymorphisms (SNPs)^10^. These regions were amplified by multiplex PCR, followed by single base extension using mass-modified dideoxynucleotide terminators of an oligonucleotide primer which anneals immediately upstream of the polymorphic site of interest. MALDI-TOF mass spectrometry then identifies the SNP allele based on the distinct mass of the extended primer from which the initial methylation status can be inferred.

***Primer design*.** DNA flanking sequences 200bp either side of signature CpG input sequences were modified to ensure suitability for assay design using the Agena Design Suite software (https://www.agenacx.com). Signature loci CpGs were modified to recapitulate *in silico* C/T SNPs introduced by methylation-dependent bisulfite conversion (Agena design tools; www.agenacx.com). Proximal SNPs and CpGs were masked to exclude them as suitable areas for primer design. To maximise amplicon recovery from potentially poor quality, degraded DNA, the optimal amplicon size was set to 120bp with a minimum of 80bp and a maximum of 200bp. In order to avoid overlaps with extension products in the mass spectrum and to facilitate multiplexing, a 10mer tag (5’ – ACGTTGGATG – 3’) was added 5’ to each PCR primer. Extension primers were 5’ tailed with variable length non-homologous sequences to generate mass differences and allow for discrimination between extension products. Rejected loci as a result of a proximal SNP with a minor allele frequency >1% or additional CpG sites impeding primer design were rescued by the use of inosine at relevant base positions. To avoid non-specific annealing, incorporated inosines were at least 3 bases away from the targeted CpG site and not more than 2 inosines per primer were permitted. Finally, the specificity of PCR primers was verified using BiSearch^28^.

***In vitro primer validation.*** The methylation status of the 17 signature loci were assayed in multiplex (Plex 1-3 as indicated; **Supplementary Fig. 2**). Plex 1 contains an additional bisulfite conversion control (Plex 1-8, bisulfite ctrl), which is invariably unmethylated and undergoes complete conversion to uracil. The three multiplexes were validated *in vitro* using a mixture series of control DNAs ranging from 0 to 100% methylation in 10% increments, using fully unmethylated and methylated control DNA samples (EpigenDX, Hopkinton, MA, USA). Bisulfite treatment of DNA using EZ DNA Methylation Kit (ZYMO Research, Irvine, CA, USA) and iPLEX PRO biochemistry (Agena Bioscience, San Diego, CA, USA) were performed according to the manufacturers’ protocols. Samples were spotted onto a 384 SpectroCHIP using an RS1000 Nanodispenser, and measured on an MA4 mass spectrometer (Agena Bioscience, San Diego, CA, USA). Mass-spectra were acquired by dedicated SpectroAcquire software, and MassARRAY Typer software was used for data analysis. Triplicate experiments were performed for each sample mixture. The methylation status of each locus (β-value) is represented by the intensities of the C allele peak. The correlation between the input and the output (% methylated) was calculated and amplicons with a poor correlation were discarded and replaced with a new CpG locus as part of the iterative redesign process **(Fig. 1a).** All signature loci had good linear correlation between the actual methylated DNA (x-axis) and the measured β-value (y-axis) with an average correlation coefficient R^2^=0.86.

***DNA Extraction*.** High molecular weight DNA was obtained from frozen tissue using the Qiagen DNeasy blood and tissue kit (Qiagen GmbH, Düsseldorf, Germany). DNA extractions from FFPE tumour sections were performed using the QIAamp DNA FFPE tissue kit or the QIAamp DNA Mini Tissue Kit (Qiagen GmbH) according to manufacturer’s instructions. For FFPE cytospin-derived nuclear preparations, a wax ring was drawn onto the glass slide around the nuclei using an ImmEdge™ wax pen. 30µl of ATL buffer was added, then a flat-edged sterile, disposable scalpel (Swann Morton Ltd.) was used to mechanically detach nuclei from the slide to create a suspension of nuclei. Subsequently, manufacturer’s instructions (QIAamp DNA FFPE tissue kit) were followed. Extracted DNA was stored at -80°C.

***DNA Quantification & Quality Measurement*.** DNA concentrations were measured using a Nanodrop 1000 Spectrophotometer (Thermo Scientific) and Qubit 2.0 Fluorometer (Invitrogen) using the dsDNA BR Assay Kit. DNA quality was assessed via the Nanodrop 260/280nm ratio. Entry criteria for validation cohort samples were 1) DNA quantity >100ng (spectrophotometry) and 2) 260/280nm ratio >1.7.

***Bisulfite conversion and QC, multiplex signature loci and iPLEX extension PCR*.** Where possible, 100ng of DNA (as measured by spectrophotometry) was bisulfite converted and purified using the Qiagen EpiTect Bisulfite kit, according to manufacturer’s protocol. To confirm successful bisulfite conversion, a QC test PCR was carried out using primers designed for fidelity to only bisulfite converted DNA, using the AmpliTaq Gold kit (Applied Biosystems, #4311806). The amplicon size was 200bp. Reaction mixtures and thermal cycling parameters are shown in **Supplementary Table 3**. In the case of negative results (assessed by standard gel electrophoresis), samples were repeated once before being classified as having failed bisulfite conversion **(Fig. 4e)**. Reaction mixtures and thermal cycling parameters used to amplify the 17 signature loci in multiplex are shown in **Supplementary Table 4**. Successful amplification was confirmed by gel electrophoresis. The general procedure for the multiplex primer iPLEX extension assay has been described^23^. Briefly, unincorporated dNTPs in multiplex signature CpG loci PCR products were degraded using 1 part shrimp alkaline phosphatase to 5 parts PCR product. This reaction mix was incubated for 40 min at 37°C followed by 5 min at 85°C and subsequently amplified using iPLEX Pro-reagent set (Agena Bioscience #10141; reaction mixtures and thermal cycling parameters used shown in **Supplementary Table 5**). Desalting was achieved by using iPLEX Pro resin (SpectroChip II and Resin kit, Agena Bioscience #10141). Primer sequences for multiplex signature loci PCR and iPLEX extension PCR are shown in **Supplementary Table 6**.

***Mass spectrometry*.** Mass spectra were acquired on a linear MALDI-TOF mass spectrophotometer (Voyager DE; PerSeptive Biosystems) workstation. The instrument is equipped with a 337nm nitrogen laser, and has a nominal ion flight path length of 1.2m. Desalted iPLEX extension reaction products were mixed 1:1 with matrix solution containing 50mg/ml 3-hydroxy picolinic acid, 0.05M ammonium citrate, and 30% acetonitrile, and were spotted in 400nl volumes onto polished (no wells) stainless steel sample plates or 400 well Teflon masked sample plates. Spectra were acquired in positive ion, delayed extraction mode. Typically, time-of-flight data from 20–50 individual laser pulses were recorded and averaged on a transient digitizer, after which the averaged spectra were automatically converted to mass by data processing software.

**Classifier design and validation.**

Support vector machine (SVM) as a supervised learning model was used for the classification task due to its good generalization ability in subgrouping. Two nonlinear multi-class SVM classifier models were used to assign subgroup and corresponding a-posteriori probability^24^ using i) the 10,000 most variably methylated CpG loci and ii) a 17-CpG locus signature from the training cohort (Fig. 1c). Linear, polynomial, sigmoid and Gaussian kernels were tested and the SVM models with the best performance were selected, as determined by use of quadratic optimization. For both classifier models (450k and MS-MIMIC), Gaussian radial basis function (RBF) kernels were used in which its parameters (i.e., gamma (γ) and cost (
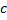
)) were optimised by applying a grid search over the two parameters in a 10-fold cross validation approach for 450k and MS-MIMIC training sets separately^24^. To train the 450k classifier, the 10,000 most variably methylated CpG loci were projected into the metagene space with 4 metagenes using the NMF technique^19^. To train the MS-MIMIC classifier, 450k estimates of methylation for the signature CpG loci were used from the training cohort. To assess the MS-MIMIC classifier, a 10-fold cross validation technique in conjunction with a random resampling was performed and predictive accuracy (area under curve) was assessed compared to the 450k classifier model. Following 1000 rounds of a 10-fold cross-validation, a 98% (with a 95% confidence interval (98.1%-99.5%)) prediction accuracy was achieved. 101/106 validation cohort samples were used to assess MS-MIMIC concordance at the level of 1) molecular subgroup call (**Fig. 1d**) and 2) estimates of methylation β-value (**Fig. 1e**). For the former, 91 samples had both 450k DNA methylation array and MS-MIMIC data. For the remaining 10 samples, subgrouping calls were made by either GoldenGate DNA methylation microarray^13^ (7%, 7/101) or confirmed *CTNNB1* mutation (WNT only; 3%, 3/101).

**Multiple imputation modelling.**

It is anticipated that when using poor quality DNA (e.g. FFPE-derived), some loci will fail to be assayed. To define the extent of, and address the handling of, missing MS-MIMIC data, the validation cohort was interrogated **(Supplementary Fig. 3)**. Firstly, a QC threshold for the acceptable number of missing β-values was identified using 10,000 randomly bootstrapped datasets (without replacement). These datasets include random missing patterns from combinations of one to ten missing β-values for each sample, where missing β-values were replaced with confounding methylation values **(Supplementary Fig. 4a, 4b)**. The prediction accuracy and confidence intervals of the MS-MIMIC classifier were obtained for these datasets **(Supplementary Fig. 4c)** and, as a result, a threshold for the maximum acceptable number of missing β-values was conservatively set to 6. Then, a bootstrap-based expectation maximization (EM) algorithm^29-31^ was used to impute missing β-values. Briefly, *m* cohorts with the same dimensions as the original cohort were firstly bootstrapped to simulate estimation uncertainty (*m* values for each missing) and then the missing values were imputed from posterior distributions obtained from running the standard EM algorithm. The *m* complete cohorts were finally combined using an averaging strategy (where *m*=20) to provide the complete MS-MIMIC dataset. To further validate the multiple imputation modelling, we examined another technique (multivariate imputation by chained equations – MICE^32^) to impute the missing β-values. There was a strong correlation between the two multiple imputation modelling methods when imputing the missing β-values in the validation cohort **(Supplementary Fig. 7)**. **Supplementary** **Table 7** shows the concordance in predicting subgroups using MS-MIMIC when using these two methods for imputing missing β-values.

**Molecular subgroup classification.**

Probability estimates of subgroup membership were determined by applying the MS-MIMIC classifier to the validation cohort. Confidence intervals were calculated by 1000 resamples of 80% of the training cohort. For the validation cohort, subgroup assignments were compared against the 450k classifier model.

**Derivation of a QC threshold for confident subgroup classification.**

To define a threshold for the identification of non-classifiable samples, the distribution of subgroup classification probability estimates was plotted and a beta distribution fitted using the moment matching estimation technique (MME)^33^. The empirically determined threshold (probability estimate=0.69) was defined as the 5^th^ percentile of the beta distribution, below which samples were non-classifiable **(Supplementary Fig. 5)**.

**MS-MIMIC classifier web application.**

The developed classifier was implemented in the reactive R shiny web framework^34^. This allows for the uploading of raw MS-MIMIC data as a comma separated value (CSV) file for medulloblastoma subgroup classification. The derived β-values and graphical representations of classification results can be downloaded. The MS-MIMIC web app can be found at [http://medulloblastomadiagnostics.ncl.ac.uk](http://medulloblastomadiagnostics.ncl.ac.uk/) **(Supplementary Fig. 6)**.

**Supplementary methods references.**

25. R Core Team (R Foundation for Statistical Computing, Vienna, Austria, 2016).

26. Frank, E., Hall, M., Trigg, L., Holmes, G. & Witten, I.H. Data mining in bioinformatics using Weka. Bioinformatics 20, 2479-2481 (2004).

27. Kuncheva, L.I. Combining Pattern Classifiers: Methods and Algorithms, Edn. 2nd, 112-117 (John Wiley and Sons 2004).

28. Tusnady, G.E., Simon, I., Varadi, A. & Aranyi, T. BiSearch: primer-design and search tool for PCR on bisulfite-treated genomes. Nucleic Acids Research 33, e9 (2005).

29. Dempster, A.P., Laird, N.M. & Rubin, D.B. Maximum likelihood from incomplete data via the EM algorithm. Journal Of The Royal Statistical Society 39, 1-38 (1977).

30. Honaker, J., King, G. & Blackwell, M. Amelia II: A Program for Missing Data. Journal of Statistical Software 47, 1-47 (2011).

31. Honaker, J. & King, G. What to do about 511 missing values in time series cross-section data. American Journal of Political Science 54, 561-581 (2010).

32. van Buuren, S. & Groothuis-Oudshoorn, K. mice: Multivariate Imputation by Chained Equations in R. Journal of Statistical Software 45, 1-67 (2011).

33. Delignette-Muller, M.L. & Dutang, C. fitdistrplus: An R Package for Fitting Distributions. Journal of Statistical Software 64, 1-34 (2015).

34. RStudio team. shiny: Easy web applications in R (2014). http://shiny.rstudio.com/
